# In Vitro Resistance-Predicting Studies and In Vitro Resistance-Related Parameters—A Hit-to-Lead Perspective

**DOI:** 10.3390/ph17081068

**Published:** 2024-08-15

**Authors:** Joanna Krajewska, Stefan Tyski, Agnieszka E. Laudy

**Affiliations:** 1 Department of Environmental Health and Safety, National Institute of Public Health NIH—National Research Institute, 00-791 Warsaw, Poland; jkrajewska@pzh.gov.pl; 2 Department of Pharmaceutical Microbiology and Laboratory Diagnostic, National Medicines Institute, 00-725 Warsaw, Poland; s.tyski@nil.gov.pl; 3 Department of Pharmaceutical Microbiology and Bioanalysis, Medical University of Warsaw, 02-097 Warsaw, Poland

**Keywords:** antimicrobial agents, antimicrobial resistance, resistance-related parameters, mutant selection, MPC, hit-to-lead stage, ALE

## Abstract

Despite the urgent need for new antibiotics, very few innovative antibiotics have recently entered clinics or clinical trials. To provide a constant supply of new drug candidates optimized in terms of their potential to select for resistance in natural settings, in vitro resistance-predicting studies need to be improved and scaled up. In this review, the following in vitro parameters are presented: frequency of spontaneous mutant selection (FSMS), mutant prevention concentration (MPC), dominant mutant prevention concentration (MPC-D), inferior-mutant prevention concentration (MPC-F), and minimal selective concentration (MSC). The utility of various adaptive laboratory evolution (ALE) approaches (serial transfer, continuous culture, and evolution in spatiotemporal microenvironments) for comparing hits in terms of the level and time required for multistep resistance to emerge is discussed. We also consider how the hit-to-lead stage can benefit from high-throughput ALE setups based on robotic workstations, do-it-yourself (DIY) continuous cultivation systems, microbial evolution and growth arena (MEGA) plates, soft agar gradient evolution (SAGE) plates, microfluidic chips, or microdroplet technology. Finally, approaches for evaluating the fitness of in vitro-generated resistant mutants are presented. This review aims to draw attention to newly emerged ideas on how to improve the in vitro forecasting of the potential of compounds to select for resistance in natural settings.

## 1. Introduction

Increasing antimicrobial resistance (AMR) is widely recognized as one of the greatest threats to humankind, and the urgent need for novel, innovative antibiotics targeting priority pathogens has been frequently emphasized for decades [1,2,3,4,5,6,7,8]. Meanwhile, not only has an insufficient number of such antibiotics entered clinics recently, but their number in the clinical pipeline is also unsatisfactory [6,9]. Behind that crisis is the high monetary cost and the lengthy time required to market a new antibiotic, combined with a high risk of its short clinical lifespan due to resistance development [6,10]. To reverse this trend, advances in preclinical resistance-predicting studies are needed [10,11,12].

Resistance may arise from mutations or horizontal gene transfer (HGT) [11,13,14]. In the first case, depending on the drug and its concentration, high-level resistance may be acquired either due to a single, spontaneous mutation (so-called single-step resistance) or due to the accumulation of several mutations, each providing low-level resistance (so-called multistep resistance) [11,13]. High-level single-step resistance usually results from a point mutation in a drug target-encoding gene (e.g., *gyrA* or *parC* in the case of fluoroquinolones or *rpoB* in the case of rifampicin). Multistep resistance may be associated with the accumulation of various non-drug target mutations (leading to increased efflux or decreased influx of a drug) and/or drug target mutations, as in the case of fluoroquinolones and *Streptococcus pneumoniae* [11,13,15]. The majority of resistance genes observed among pathogens are acquired via HGT, involving one of three main mechanisms: transformation with free DNA, transduction by bacteriophages, or conjugation [11,14,16]. The risk of HGT-mediated resistance evolution is especially high in high-density and genetically complex communities (e.g., in the gastrointestinal tract) in the presence of selective pressure [11]. However, only a small percentage of emerging mutants have the ability to become fixed in the population and subsequently spread [11,16]. This depends on the fitness costs of resistance, the antibiotic concentration in the environment, and a plethora of other external conditions, such as the host’s immune system activity [17,18,19,20,21]. For each drug-strain pair, a range of selective concentrations exists within which resistant mutants are enriched, regardless of how and when they have emerged [15,18,19,22]. This range extends from the minimal selective concentration (MSC), at which the growth rate of mutants and wild-type cells is equal, to the mutant prevention concentration (MPC), at which the growth of the most resistant mutant in the population is inhibited. The concentration range between the MSC and MIC of wild-type cells is called the sub-MIC selective window, whereas the concentration range between the MIC and MPC is called the mutant selection window (MSW) [15,18,19,22]. Exposure of microorganisms to sub-MIC selective concentrations occurs frequently both in the environment and during antibiotic therapy, e.g., due to inadequate dosing or poor drug penetration into specific tissues and organs [19,22,23,24,25,26]. Exposure to concentrations from the MSW ranges may still occur during therapy since MIC values remain the exclusive basis of current dosing regimens, i.a., due to the fact that MPC values frequently exceed the toxicity threshold while dosing above the MIC is sufficient for therapeutic success in most cases [15,27]. However, when the bulk of therapies worldwide place antibiotic concentrations within their MSW, the abundance of selected mutants accelerates the loss of their activity [15,27]. Assuming that the selective concentration range depends on the structure of the compound, it is possible to optimize drug candidates in those ranges via structural modifications [18,27,28,29,30,31,32,33]. Ideally, such optimization should take place at the hit-to-lead stage to further develop compounds with high activity, to which evolutionary pathways leading to resistance are constrained and can additionally be safely administered above their MPCs [27,34]. 

At the hit-to-lead stage, evaluating the antimicrobial activity of compounds via MIC determination is well-standardized [35,36], but no such gold standard exists to evaluate their potency for selecting resistance. Predominantly, for selected hits, the frequency of spontaneous mutant selection (FSMS) is calculated [37,38,39,40,41,42,43,44], supplemented by the MPC determination [41,43,44,45,46] or various adaptive laboratory evolution (ALE) experiments under drug pressure aimed to evaluate the level (i.e., the x-fold MIC increase), time, and mutations required for multistep resistance to emerge [34,37,42,43,44,45,47,48]. However, resistance-related in vitro parameters derived from these studies are still considered to have unsatisfactory predictive values for further development [10,11,12]. For instance, single-step mutants resistant to some already marketed drugs such as methicillin or fosfomycin are frequent in vitro but rare in vivo [49], whereas a benzoxaborole GSK2251052 (now known as epetraborole) failed in phase 2 clinical trials for the treatment of complicated urinary tract infections despite an acceptable FSMS in vitro [41,50]. MPC values, apart from unsatisfactory in vivo reproducibility [49,51], were also reported to display poor experiment-to-experiment repeatability [52]. Finally, a variety of ALE approaches that are used to generate in vitro multistep-resistant mutants (i.e., serial transfer of batch cultures, continuous culture, and evolution in spatiotemporal microenvironments) are known to yield different outcomes [21]. Thus, the question remains: which ALE approach or approaches should be implemented at the hit-to-lead stage to accurately predict compound potency to select multistep resistance in natural settings? Even acknowledging the fact that in vitro studies cannot fully mimic natural conditions due to the intrinsic lack of host factors (especially immune system activity), there is a need to improve such experiments and increase their predictive value in order to limit subsequent animal studies [11]. Moreover, conventional ALE protocols are usually reagent-consuming and require considerable hands-on time for up to several weeks. This impedes the widespread performance of such experiments in the hit-to-lead stage, where the availability of compounds is often limited, and many derivatives should be investigated within a reasonable timeframe to provide a constant supply of new drug candidates for further development.

However, new resistance-related in vitro parameters have recently emerged (i.e., dominant mutant prevention concentration—MPC-D, inferior-mutant prevention concentration—MPC-F) [53]. Moreover, new approaches and high-throughput setups for ALE experiments have been developed [54,55,56,57]. This review presents in vitro resistance-related parameters that can be determined at the hit-to-lead stage (FSMS, MPC, MPC-D, MPC-F, and MSC), applicable methodologies (including modifications enabling their use for the assessment of HGT-mediated resistance evolution), and their predictive value for further development. The utility of ALE approaches employing serial transfer, continuous culture, or evolution in spatiotemporal microenvironments for comparing hits in terms of the level and time required for multistep resistance to emerge is considered. We also discuss how the hit-to-lead stage can benefit from various high-throughput, automized, miniaturized, and low-cost ALE setups developed over the years, like robotic workstations for serial transfer [58,59,60], do-it-yourself (DIY) continuous cultivation systems [54,61,62,63,64,65,66,67,68,69,70], microbial evolution and growth arena (MEGA) plates [71,72,73,74], soft agar gradient volution (SAGE) plates [55], microfluidic systems [56,75,76,77], and microdroplet technology [57]. Finally, approaches to evaluate the fitness of in vitro-generated resistant mutants have been presented, including competition experiments in complex microbial communities [78,79,80]. The aim of this review is to draw attention to newly emerged ideas on how to improve and accelerate in vitro forecasting of the potential of compounds to select for resistance in natural settings. 

## 2. Single-Step Resistant Mutant Selection Studies and Derived Parameters

### 2.1. Frequency of Spontaneous Mutant Selection (FSMS)

Single-step, spontaneous mutants can be selected in vitro by applying 10^8^–10^11^ CFUs on agar plates containing incremental concentrations of a drug, usually ranging from the MIC value to 8–64 × MIC. Following incubation, the obtained colonies are restreaked on plates with the same drug concentration, and colonies able to regrow are considered resistant mutants [37,38,39,40,41,42,43,44,53]. This regrowth testing is necessary to eliminate the so-called inoculum effect leading to the occurrence of false mutants, i.e., colonies exhibiting the wild-type MIC. They frequently appear in such experiments because high-density inocula protect them from the drug’s action [81,82]. This effect can also be eliminated by applying fewer cells to more plates [30]. However, this approach, which is time- and substance-consuming, is difficult to implement in preclinical screening. Finally, for each concentration, the FSMS can be calculated as the ratio of resistant CFUs to the number of CFUs applied on the plates [53,83,84,85,86,87]. Concentrations with a frequency below 1 × 10^−8^ CFU/mL are sometimes considered a threshold for the reduced mutant selection, e.g., in a study by Sun et al. assessing meropenem–vaborbactam activity against *Klebsiella pneumoniae* carbapenemase (KPC)-producing strains [83]. Such an approach is based on the fact that mutants arising at a frequency of 1 × 10^−6^ to 1 × 10^−8^ can be controlled by the hosts’ immune systems [18,27]. However, even mutants that rarely emerge can be selected among immunocompromised patients, which paves the way for their subsequent spread [18]. Therefore, dosing regimens that can completely block the growth of single-step mutants were proposed as a strategy to prolong the lifespan of antimicrobials [18].

### 2.2. Mutant Prevention Concentration (MPC)

**Figure 1:**
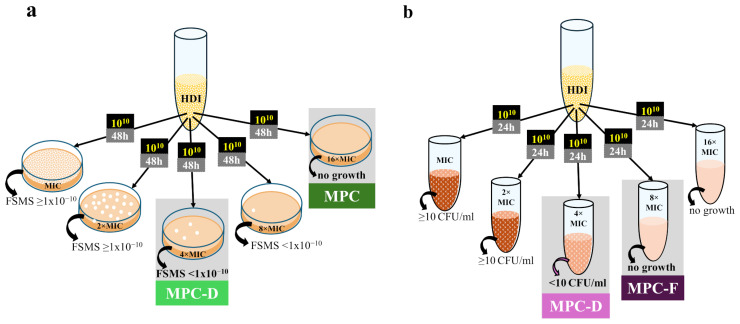
General procedures for the determination of in vitro parameters related to single-step resistant mutants: (**a**) Agar dilution method; (**b**) Broth dilution method. HDI, high-density inoculum; FSMS, frequency of spontaneous mutant selection; MPC, mutant prevention concentration; MPC-D, dominant mutant prevention concentration; MPC-F, inferior-mutant prevention concentration.

The term MPC was coined by Dong et al. in 1999 and is defined as the lowest concentration of an antibiotic that completely inhibits the growth of microorganisms when at least 10^10^ CFU is applied to agar plates (Figure 1a) [28]. Such a large population is likely to contain spontaneous mutants since FSMS values are usually 10^−6^–10^−8^. It is also a population size typical for many infection sites [15,28]. It was noted that when dosing regimens exposed microorganisms to concentrations within the MSW range, resistance was observed relatively quickly after drug marketing [18,27]. In turn, when antibiotic concentrations during therapy exceed MPC values, resistant strains are rarely isolated in clinics [30,32]. MPC determination can also be modified for application to HGT-related resistance situations by adding a small number of resistant mutants to the susceptible population for relevant measurements [88,89].

However, the proposed methodology presents several challenges. First, an inoculum effect occurs during such experiments and cannot be entirely avoided, even in a liquid environment [81,82]. Thus, studies aiming to determine the MPC via broth microdilution utilized inocula with lower densities [90,91], whereas the original agar dilution method remains laborious and reagent-consuming. Second, Gianveccio et al. reported poor experiment-to-experiment repeatability of the MPC values obtained within the same laboratory [52]. Third, discrepancies between the in vitro and in vivo determined MSWs occurred, mainly linked to decreased mutant fitness, which is not being evaluated directly during MPC determination [49,51]. This prompted us recently to propose a new approach to determine the upper boundary of the MSW [53].

### 2.3. Dominant Mutant Prevention Concentration (MPC-D) and Inferior-Mutant Prevention Concentration (MPC-F)

Since extremely rare selected mutants may not always be detectable by the agar dilution method, in an attempt to increase the repeatability of the in vitro-determined MSW ranges, we recently proposed that the FSMS value should provide a threshold for its upper boundary [53]. Assuming that at least 1 × 10^10^ CFU should be tested to determine the MPC1010, we propose the lowest concentration with the FSMS < 1 × 10^−10^ as the new parameter called MPC-D, marking the upper boundary of the dominant mutant selection window (MSW-D) (Figure 1a) [53]. However, to better assess whether drug-resistant mutants selected in vitro are significantly less fit than wild-type cells, we have proposed the broth dilution method to determine the MPC-D value (Figure 1b) [53]. In this method, we defined the MPC-D as the lowest drug concentration that prevents drug-resistant mutants selected from among 10^10^ CFU from establishing a resistant population with a density of at least 10 CFU/mL during 24 h of incubation in a liquid medium with the compound. In turn, the lowest concentration that completely inhibited mutant growth in this method was named MPC-F. It refers to mutants with high fitness costs of resistance, which can arise in vitro, but assuming their inability to dominate the population, they are unlikely to also be selected in vivo [53]. In our study, the MPC-D of ciprofloxacin for *Staphylococcus aureus* determined via the broth dilution method was lower than its MPC-F. Thus, the proposed broth dilution method allows for the differentiation of dominant mutants from mutants with impaired fitness, which may decrease the discrepancies between in vitro and in vivo values [53].

## 3. MultiStep-Resistant Mutant Selection Studies

### 3.1. Serial Transfer of Batch Cultures

Multistep-resistant mutants are usually generated in vitro during ALE experiments by employing the serial transfer approach. This approach involves regularly transferring a portion of a culture to a fresh medium at established intervals (e.g., daily or after growth becomes visible) [21]. Consequently, periodic variations in environmental conditions occur since the nutrient availability, population size, and growth rate alternate between high and low values as the culture is diluted. Thus, evolving populations experience bottlenecks and feast-and-famine regimes, which may mimic, e.g., the vertical transmission of pathogens, where each affected host could be regarded as a new batch culture. However, the serial transfer approach poses the risk that some mutants may be accidentally lost due to a bottleneck [21]. 

**Figure 2:**
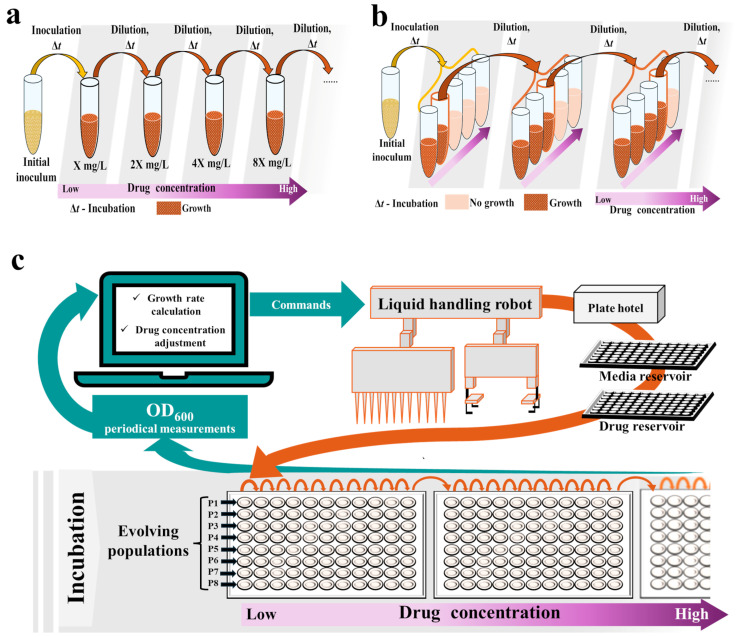
Setups for adaptive laboratory evolution (ALE) experiments employing the serial transfer approach: (**a**) Conventional setup for the drug increment approach; (**b**) Conventional setup for the drug gradient approach; (**c**) Robotic workstation. OD, optical density.

Two main types of serial transfer experiments are the most prevalent: (I) the drug increment approach, where the concentration of a drug is increased progressively, usually by 1.5–2-fold at each dilution (Figure 2a) [92,93,94,95,96,97,98], and (II) the drug gradient approach, where cultures from the highest drug concentration with visible growth are transferred again to the whole gradient of concentrations at fixed intervals (Figure 2b) [86,99,100,101,102,103,104,105,106]. Alternatively, serial transfer at constant subinhibitory drug concentrations can be performed [107,108,109]. Conducted by Jahn et al., a comparison of the geno- and phenotypes of *Escherichia coli* after evolution to amikacin, piperacillin, and tetracycline under drug gradient and drug increment regimes revealed that the key mutations conferring drug resistance arise regardless of the applied approach. However, mutants selected under the mid-pressure of the drug gradient regime displayed an improved growth rate [110].

Assuming the stochastic nature of evolutionary changes while employing the serial transfer approach, many independent culture lines should be run concurrently for reliability. This increases the hands-on time required for daily manipulations, as well as the usage of tested compounds and other reagents [21]. Thus, although serial transfer can be performed manually with standard laboratory equipment such as flasks or tubes, various attempts have been made to miniaturize and automate these experiments, e.g., by conducting passages on microtiter [59,100,105,109,111] or deep-well plates [98,112], and employing either manual replicators [107] or robotic workstations consisting of a PC, a liquid-handling robot, a shaking incubator, a microtiter plate reader, and a microplate hotel (Figure 2c) [34,58,59,60,108,112,113]. In automated serial transfer setups, microtiter plates carrying cultures are incubated in a shaker, and the optical density (OD) is measured at fixed intervals [34,58,59,113]. From the OD measurements, the growth rate of each culture is calculated and compared with the growth rate determined in the absence of the antibiotic. Once it exceeds a fixed threshold (i.e., 30% of the no-antibiotic culture growth rate), cultures are automatically diluted with fresh media in the next wells, whereas the remaining portion of the culture is automatically mixed with glycerol and frozen. The antibiotic concentration can also be automatically adjusted at each dilution, e.g., if the growth rate exceeds 50–75% of the no-antibiotic culture growth rate, the concentration in the following passage is increased by a factor of 1.5–2; otherwise, it is maintained. This procedure keeps cells growing under sustained selective pressure at growth rates close to 50% of those of non-inhibited cells [34,58,59,113]. Such miniaturization and automation of serial transfer limit the risk of human error, reduce the hands-on time required, improve environmental control, and increase experimental throughput—even up to hundreds of populations can be conducted simultaneously [34,58,59,103,104,113]. Such properties are desirable during preclinical screening, although setting up and running a robotic workstation requires space and trained personnel. It also remains relatively expensive.

### 3.2. Continuous Culture

**Figure 3:**
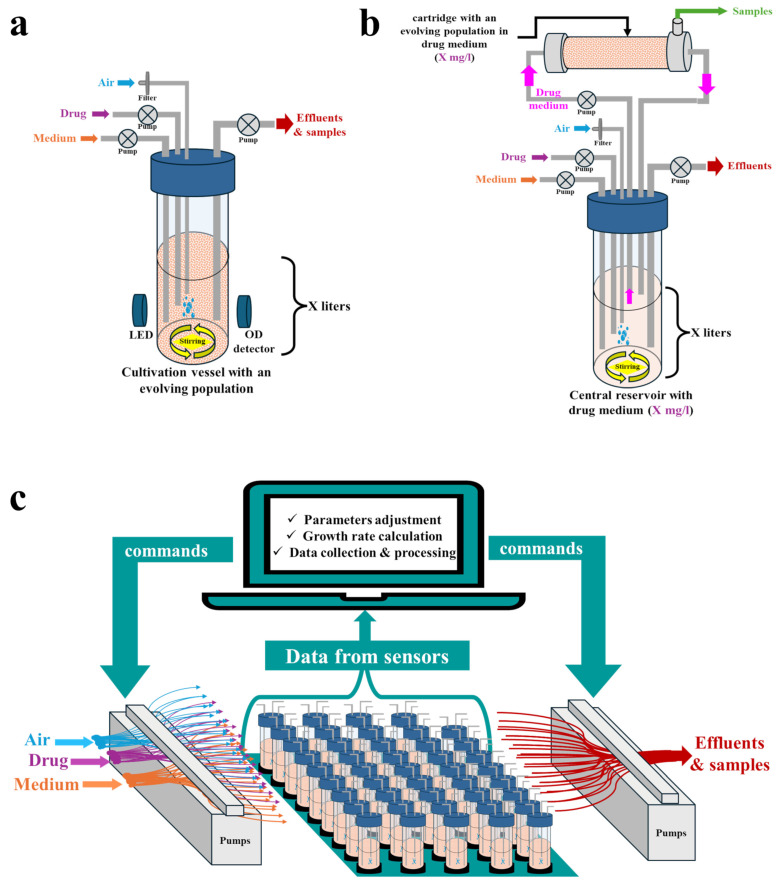
Setups for adaptive laboratory evolution (ALE) experiments employing a continuous culture approach: (**a**) Conventional one-compartment system (chemostat); (**b**) Two-compartment hollow fiber infection model (HFIM); (**c**) High-throughput, do-it-yourself (DIY) system.

In the continuous culture approach, microbes are continuously cultivated under precisely controlled and well-mixed conditions within a closed vessel (bioreactor) [21]. An influent pump continuously supplies the bioreactor with a fresh medium, while a second pump removes liquid from the vessel at the same rate. Thus, the volume inside is kept constant (Figure 3a). In theory, continuous culture systems enable the infinite and automated maintenance of the culture under precisely controlled and constant conditions without bottlenecks and feast-and-famine regimes typical of serial transfer. It also offers the possibility of repeated sampling for microbiological and biochemical studies without disrupting the stability of the culture [21]. It was shown that mutants of the same parent strains obtained via continuous culture display increased growth rates compared to those that evolved during serial transfer experiments [97,114]. Moreover, mutations leading to resistance may differ between these approaches, as in the case of *Enterococcus faecium* and daptomycin [97,114].

#### 3.2.1. Types of Cultivation Vessels

Various types of continuous culture systems have been employed to track antimicrobial resistance evolution in vitro. In chemostats, fresh medium is continuously supplied at a fixed dilution rate, while the microbial culture is simultaneously removed at an equal rate [21,115]. This enables the tracking of resistance evolution under conditions simulating either defined dosing regimens [116,117] or various environments, e.g., a water treatment plant [118]. However, chemostats unavoidably lead to dilution of the culture, which may result in the loss of some resistant sub-populations [115]. To avoid this, continuous culture can be performed using a two-compartment hollow fiber infection model (HFIM) (Figure 3b) [115,119]. In this model, fresh medium is supplied to a central reservoir (without microbes), from which it is circulated to the peripheral hollow fiber cartridge, where microbes are entrapped in the extra capillary space. Waste products from the cartridge are circulated back to the central reservoir, from where they are finally eliminated. The drug concentrations in the central reservoir and in the peripheral cartridge remain equal [115,119]. At the hit-to-lead stage, chemostats or HFIMs can be employed to validate whether spontaneous or HGT-obtained resistant mutants indeed emerge when drug concentrations oscillate within the MSW boundaries determined by the original agar dilution method [89,120,121,122,123,124]. 

Bioreactors can also function as turbidostats by continuously monitoring the culture density and automatically adjusting the dilution rate to maintain constant turbidity at a defined level. They can be used to track resistance evolution under subinhibitory drug concentrations, or the drug concentration can be increased manually during the experiment [97,114]. In contrast, in morbidostats, the dilution rate is fixed throughout the experiment, and an algorithm dynamically adjusts the drug concentration at each dilution in response to microbial growth, such that approximately 50% of the growth inhibition of an evolving population is maintained [65,125]. This enables the tracking of evolutionary pathways leading to high-level resistance and their reproducibility among populations evolving in parallel. For instance, Toprak et al. tracked *E. coli* resistance evolution to chloramphenicol, doxycycline, and trimethoprim over a period of ~20 days, cultivating five isogenic populations in parallel for each drug [125]. Resistance levels increased dramatically for all drugs, with parallel populations showing similar phenotypic trajectories [125]. Yoshida et al. used this system to track *E. coli* resistance evolution under cyclic exposure to various antibiotic pairs consisting of kanamycin, polymyxin, chloramphenicol, nalidixic acid, and nitrofurantoin [126].

#### 3.2.2. Do-It-Yourself (DIY) Setups

Commercially available bioreactors and single-use cartridges for HFIMs remain relatively expensive and usually operate in large volumes, thereby consuming high amounts of reagents and tested compounds [21]. The high cost and low throughput impede the widespread application of such systems at the hit-to-lead stage. However, attempts have been made to create miniaturized, low-cost DIY continuous culture systems exploiting either standard laboratory equipment or 3D-printed parts, with the possibility of running many individually controlled and logged bioreactors concurrently (Figure 3c). Several detailed, step-by-step protocols and video instructions for building such systems are available [54,61,62,63,64,65,66,67,68,69,70], along with dedicated, open-source software [54,61,63,64,65,68,69,70,127]. Some of them are suitable for the culture of biofilms [128], whereas others can be constructed with minimal engineering and programming experience [70]. The most complex and highly configurable DIY setup for continuous culture thus far is eVOLVER, which was proposed by Wong et al. [54]. It consists of highly modular, open-source wetware, hardware, electronics, and web-based software with a high potential for parallelization (up to hundreds of populations can be cultivated and monitored concurrently). eVOLVER can be programmed to monitor any parameter chosen by the operator and can function as a chemostat, turbidostat, or morbidostat. Moreover, each culture vessel (a glass vial) can be run with a different medium, temperature, and culture agitation rate [54,69]. For example, it was recently used as a chemostat, simulating wastewater conditions to track the evolution of *E. coli* resistance toward various drug combinations [129], whereas Langevin et al. used eVOLVER operating as a turbidostat to determine under which concentrations (within the range of 0.25–10 × MIC) three *E. coli* strains can evolve resistance to chloramphenicol within 72 h and to determine the ultimate resistance level (final MIC value) for each concentration [130]. Assuming a high throughput of the eVOLVER framework, many derivatives can be compared within a reasonable timeframe in terms of the level and time required for multistep resistance to emerge. Thus, despite the challenges of assembling and operating such DIY systems, which require at least basic engineering and programming skills, they pave the way for the application of continuous culture setups in early preclinical screening.

### 3.3. Resistant Mutant Selection in Spatiotemporal Environments

**Figure 4:**
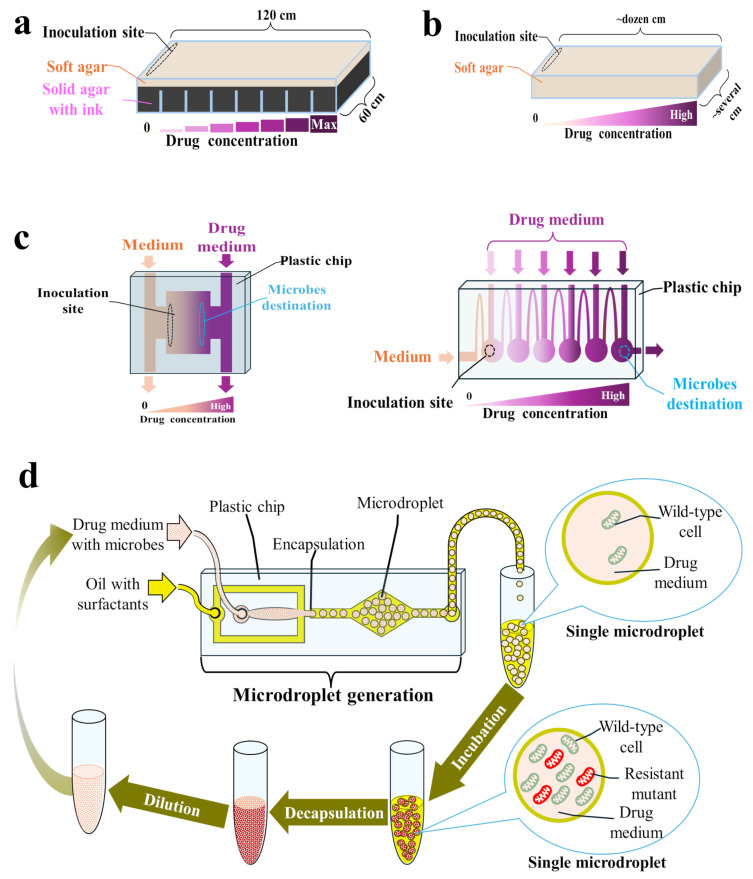
Setups for adaptive laboratory evolution (ALE) experiments in spatiotemporal microenvironments: (**a**) Microbial evolution and growth arena (MEGA) plate; (**b**) Soft agar gradient evolution (SAGE) plate; (**c**) Microfluidic chips; (**d**) Microdroplet-based system.

Both serial transfer and the continuous culture approach enable tracking of the resistance evolution of primarily planktonic populations in homogeneous and well-mixed environments [21], although some protocols to conduct ALE experiments with biofilms are also available [97,114,128,131,132]. In reality, microorganisms usually inhabit heterogeneous and structured environments, where both planktonic and biofilm populations are hosted, and are often exposed to antibiotic gradients and changing conditions. This can also be seen in the human body during antibiotic therapy, e.g., due to limited antibiotic penetration into various tissues [55,75,133]. In such environments, microbes can migrate between different niches instead of competing with their neighbors for limited resources, which may alter the evolutionary outcome [21,55,71]. It has also been proven that resistant mutants emerge faster in connected microenvironments [75]. Thus, conducting ALE experiments predominately with planktonic cultures in homogenous environments may improve the in vivo reproducibility of the selective concentration ranges determined in vitro. Therefore, to better predict the multistep resistance evolution in natural settings, alternative methodologies have been proposed, utilizing either solid or semi-solid media [55,71], microfluidic chips [75], or microdroplet technology [57]—Figure 4.

#### 3.3.1. Microbial Evolution and Growth Arena (MEGA) Plates

Baym et al. developed an experimental device called the MEGA plate, enabling the migration and adaptation of microbes across a large (120 × 60 cm), spatially structured environment [71]. This plate consists of a rectangular acrylic dish containing solid (2%) black agar regions with progressively increasing concentrations of an antibiotic, overlaid by a soft (0.28%) agar layer in which microbes can swim and migrate to the next plate region [134,135]. Soft agar is considered a good imitation of many natural settings, such as soft tissues [134,135]. During experiments on a MEGA plate, microbes are inoculated in the drug-free region. Once the nutrients in that region have run out, resistant mutants spread by chemotaxis to the next region containing available nutrients along with a drug. The large size of the plate provides space for a large population to evolve for a long time (up to 60 days) without mutants blocking each other physically [71,72]. In addition, due to its large size, the antibiotic gradient is stably maintained throughout the experiment despite diffusion [71]. During the experiment, the rate of displacement by bacteria can be measured and used as an indicator of the adaptation rate. Microbes can also be sampled from the plate for further analysis, and their spread can be video recorded [71,72]. While evolving on the MEGA plate, *E. coli* strains increased their resistance to trimethoprim (within 10 days) and ciprofloxacin (within 12 days) by 10,000-fold and 100,000-fold, respectively [71]. In another study with *E. coli*, the MICs of amoxicillin (within 5 days), cefotaxime (within 13 days), and florfenicol (within 10 days) increased 64-fold, 533-fold, and >32-fold, respectively [73,74]. Meanwhile, *Pseudomonas aeruginosa* strains sensitive to colistin (MICs < 2 mg/L) were able to inhabit MEGA plate regions containing 400 mg/L colistin within 15–60 days, displaying up to 32-fold MIC increase [72]. However, MEGA plate assay is applicable to motile strains only. Moreover, operating these devices is associated with a significant contamination risk and requires dedicated space and custom-fabricated plates.

#### 3.3.2. Soft Agar Gradient Evolution (SAGE) Plates

To make ALE experiments in spatiotemporal environments more accessible, Ghaddar et al. designed a compact system based on SAGE plates containing an antibiotic gradient prepared in a soft agar medium (0.2–0.75% agar weight/volume) [55]. SAGE plates can be prepared on basic laboratory plastics (e.g., on Petri dishes), and once inoculated, they can be incubated for up to six days, not requiring operator involvement [55]. As in the MEGA plate, inoculated microbes grow until they reach inhibitory concentrations of the antibiotic, accessible only for resistant mutants, where they can quickly establish a buffer for discarded cells, blocking competition from faster-growing but more antibiotic-susceptible cells [55,136]. Evolved mutants can be extracted from SAGE plates using a pipette [55,136]. Interestingly, *E. coli* resistance to many antibiotics evolved faster and easier on SAGE plates than on morbidostat [55]. The same was observed with antibiotics, such as ampicillin, doripenem, and polymyxin B, for which resistance evolution in clinics was relatively slow [55]. SAGE plates have also been proven to be high-throughput setups. For instance, Chowdhury et al. used them to evolve chloramphenicol-resistant mutants of *E. coli* and subsequently to evolve a total of 16 independent isogenic populations of the wild type and separately obtained mutants to two different antibiotics in parallel [136].

#### 3.3.3. Adaptive Evolution on Microfluidic Chips

Natural, heterogeneous environments with different niches can also be mimicked on microfluidic chips, i.e., on glass, silicon, or polymer plates with an etched or molded network of microchannels and microchambers [56,75,76]. Microbes can be continuously cultivated under the generated antibiotic gradient within the designed microcompartments. This gradient is created by introducing different drug concentrations at different locations on the chip and allowing them to diffuse and mix within the microfluidic channels. As in the MEGA and SAGE plates, resistant mutants actively move from regions of low drug concentration toward zones with higher concentrations. The single-use microfluidic chip is accompanied by a fluid flow controller and a PC or microscope so that the resistance evolution can be observed continuously and recorded. Following the experiment, microbes can be transferred from the chip to drug-containing plates for further analysis [56,75,76]. Such setups consume extremely small volumes of a drug and other reagents while offering a high level of autonomy and precision. Moreover, resistant mutants on microfluidic chips can be obtained within a few hours or days, depending on the design [56,75]. Such acceleration of resistance evolution results from breaking up the population into smaller, separated populations, within which resistant mutants are more likely to become fixed, as there are fewer cells to outcompete [75]. 

Chips with several layouts were used to track the resistance evolution of *E. coli* to ciprofloxacin [75,76], *Shewanella oneidensis* to ciprofloxacin and polymyxin B [137,138,139], and *E. coli* to rifampicin and nalidixic acid [56]. Moreover, microfluidic chips that are especially suitable for tracking the enrichment of antibiotic-resistant bacteria in biofilms have also been developed [77]. Among their limitations is the proneness of microchips to clogging with accumulated biomass and biofilm formation. Thus, they are not suitable for long-term experiments—most studies lasted 2–8 days [56,75]. Moreover, the design and fabrication of microchips may be cumbersome, and only a few commercially available products are available. Moreover, setting up and operating the entire system for such evolution-on-a-chip requires certain expertise and engineering skills. Nevertheless, the presented proof-of-concept studies may be considered a stepping stone toward the widespread performance of low-cost and high-throughput resistance-predicting studies during antimicrobial preclinical screening.

#### 3.3.4. Adaptive Evolution in Microdroplets

Microfluidic chips can be used to generate microdroplets, within which a strictly controlled number of cells (usually a few) are encapsulated with a drug or with a combination of several drugs [57,140,141,142,143]. One milliliter of such an emulsion contains approximately 2.6 million microdroplets of 90 μm diameter, and each droplet may be considered a single chemostat. As the cells are separated, evolution in microdroplets protects resistant but slower-growing mutants from being outcompeted [57,140,141,142,143]. According to the protocol proposed by Seo et al., on the first day of the experiment, the overnight bacterial broth cultures should be diluted appropriately with a drug-containing medium and injected into the microfluidic chip along with a fluorinated oil with surfactants to generate microdroplets. The obtained microdroplets are subsequently incubated in flasks for 24 h, then chemically broken with a demulsifier, diluted in fresh drug-containing medium, and encapsulated again [57,142]. Among the mutants of *E. coli* resistant to doxycycline that evolved in microdroplets, several new, unexpected mutations were identified, apart from those also identified after serial passage experiments [142].

Disney-McKeethen et al. recently applied a similar methodology to adapt *P. aeruginosa* to increasing colistin concentrations [141]. This study aimed to track resistance evolution in conditions that mimic well the environment of a lung with cystic fibrosis (CF), which is highly heterogeneous, with many isolated regions with varying levels of nutrients, oxygen, and antibiotics. It was confirmed that the mutations associated with resistance in well-mixed batch cultures differ from those that arise in microdroplets [141], which emphasizes the importance of studying antibiotic resistance evolution in environments that simulate well the ecological conditions where resistance may arise.

## 4. Fitness Cost Evaluation and Minimal Selective Concentrations (MSCs) Determination

Once resistant mutants are obtained, their fitness with respect to the parent strains should be evaluated at various drug concentrations in the absence of a drug. This can be performed either (I) indirectly by comparing the growth rates of the evolved populations and a parent strain [144] or (II) directly by performing head-to-head pairwise competition assays involving co-culturing of the wild-type and evolved mutant strains mixed in known initial proportions and tracking their growth over time for up to 14 days—the fittest strain should increase in proportion [19,145]. During competition experiments, competing strains can be differentiated from each other. For example, Worthan et al. used the *araBAD* operon; an *E. coli* strain with a *ΔaraBAD* genotype produced dark red colonies on TA agar, whereas the strain with an intact *araBAD* operon appeared as beige or dusty pink colonies [145]. Alternatively, strains may be genetically tagged with variants of the green fluorescent protein gene (*yfp* and *cfp*, encoding yellow- and cyan-fluorescent proteins, respectively) and counted via fluorescence-activated cell sorting (FACS), which diminishes the errors associated with counting small populations [19]. 

Finally, a relative fitness (*W*) value can be calculated by dividing the natural logarithm of the ratio of final CFU/mL to initial CFU/mL for both strains and dividing the values obtained for a resistant mutant by the values obtained for a wild-type strain. A *W* > 1 implies that the resistant mutant is a better fit than its ancestor [145]. The selection coefficient (*s*) can be calculated by finding the natural logarithm of the ratio of the final CFU/mL to the initial CFU/mL for both strains, finding the difference, and dividing by the day of competition. A positive selection rate (*s* > 0) implies that the resistant mutant is fitter than its ancestor, whereas a selection rate of zero implies equal fitness over time [19,145,146]. The obtained selection coefficients can be subsequently plotted as a function of drug concentration, and the MSC value can be determined as the lowest concentration for which *s* = 0 [19,146]. 

It should be emphasized that in vitro-evaluated fitness does not necessarily equal mutant fitness in vivo due to the intrinsic lack of host immune system activity in in vitro models. Such discrepancies were observed, for instance, with the fosfomycin and *P. aeruginosa* [51], cefiderocol and *Stenotrophomonas maltophilia* [117], or cefiderocol and *Acinetobacter baumannii* [116]. Thus, in vitro fitness evaluation may represent a “worst-case scenario”. However, to increase the predictive value of such experiments, MSCs can also be determined by utilizing complex microbial communities representing various microbiomes [78,79,80]. It was found that under such conditions, MSCs are higher than in pairwise assays because the complex community protects wild-type cells, and the fitness costs of resistance are higher [78]. Regardless of the approach, ALE experiments aimed at evaluating mutant fitness and determining the MSC values employ either serial transfer [19,78,145,146] or a continuous culture approach [121]. Assuming that various automated and high-throughput setups exist for such studies, they can also be performed during the hit-to-lead phase to increase the predictive value of resistance-related studies.

## 5. Conclusions

Over the years, various ideas have emerged on how to assess in vitro which compounds can lose their clinical efficacy too quickly. Even assuming that in vitro studies cannot fully mimic natural conditions due to the lack of host factors (especially immune system activity), it was proven that they could be redesigned to better predict the selective concentration ranges of compounds in natural settings. For instance, concentrations inhibiting the growth of single-step resistant mutants can be determined by the broth dilution method, which differentiates mutants into dominant ones (i.e., those without significant fitness costs, thus likely to also be selected in vivo) and inferior ones (i.e., mutants with impaired fitness, thus unlikely to be selected in vivo) [53]. Moreover, the potency of compounds to select for multistep resistance may be evaluated in vitro in conditions tailored to mimic different natural settings, e.g., in spatiotemporal microenvironments imitating in-host conditions (e.g., created from solid or soft agar, on microfluidic chips, or in microdroplets) and in well-mixed conditions of bioreactors simulating environmental hotspots such as wastewater plants [116]. Finally, various automated, miniaturized, and high-throughput setups for ALE experiments developed recently pave the way for their broader implementation in the hit-to-lead stage. They can be employed not only to compare compounds in terms of the level and time required for the multistep de novo resistance to emerge under various conditions but also to assess the risk of HGT-mediated resistance development by performing such experiments with mixed inocula containing wild-type cells and resistant mutants. Moreover, they can improve the evaluation of mutant fitness in competition experiments, which can also be performed with mutants obtained via HGT [147] or in microbial communities [78] to increase their predictive value. Altogether, the presented ideas prove that it is possible to improve, accelerate, and scale up resistance-predicting studies conducted at the hit-to-lead stage.

## Data Availability

The data presented in this study are included in the submitted manuscript.
